# Comparative analysis of loop-mediated isothermal amplification (LAMP)-based assays for rapid detection of SARS-CoV-2 genes

**DOI:** 10.1038/s41598-021-01472-3

**Published:** 2021-11-18

**Authors:** Daniel Urrutia-Cabrera, Roxanne Hsiang-Chi Liou, Jiang-Hui Wang, Jianxiong Chan, Sandy Shen-Chi Hung, Alex W. Hewitt, Keith R. Martin, Thomas L. Edwards, Patrick Kwan, Raymond Ching-Bong Wong

**Affiliations:** 1 Centre for Eye Research Australia, Royal Victorian Eye and Ear Hospital, Melbourne, Australia; 2 Ophthalmology, Department of Surgery, University of Melbourne, Melbourne, Australia; 3 Department of Neuroscience, Central Clinical School, Monash University, Melbourne, Australia; 4 Departments of Medicine and Neurology, University of Melbourne, Royal Melbourne Hospital, Melbourne, Australia; 5 Shenzhen Eye Hospital, Shenzhen University School of Medicine, Shenzhen, China

**Keywords:** Biological techniques, Microbiology techniques

## Abstract

The COVID-19 pandemic caused by SARS-CoV-2 has infected millions worldwide, therefore there is an urgent need to increase our diagnostic capacity to identify infected cases. Although RT-qPCR remains the gold standard for SARS-CoV-2 detection, this method requires specialised equipment in a diagnostic laboratory and has a long turn-around time to process the samples. To address this, several groups have recently reported the development of loop-mediated isothermal amplification (LAMP) as a simple, low cost and rapid method for SARS-CoV-2 detection. Herein we present a comparative analysis of three LAMP-based assays that target different regions of the SARS-CoV-2: ORF1ab RdRP, ORF1ab nsp3 and Gene N. We perform a detailed assessment of their sensitivity, kinetics and false positive rates for SARS-CoV-2 diagnostics in LAMP or RT-LAMP reactions, using colorimetric or fluorescent detection. Our results independently validate that all three assays can detect SARS-CoV-2 in 30 min, with robust accuracy at detecting as little as 1000 RNA copies and the results can be visualised simply by color changes. Incorporation of RT-LAMP with fluorescent detection further increases the detection sensitivity to as little as 100 RNA copies. We also note the shortcomings of some LAMP-based assays, including variable results with shorter reaction time or lower load of SARS-CoV-2, and false positive results in some experimental conditions and clinical saliva samples. Overall for RT-LAMP detection, the ORF1ab RdRP and ORF1ab nsp3 assays have faster kinetics for detection but varying degrees of false positives detection, whereas the Gene N assay exhibits no false positives in 30 min reaction time, which highlights the importance of optimal primer design to minimise false-positives in RT-LAMP. This study provides validation of the performance of LAMP-based assays as a rapid, highly sensitive detection method for SARS-CoV-2, which have important implications in development of point-of-care diagnostics for SARS-CoV-2.

## Introduction

The outbreak of Severe Acute Respiratory Syndrome Coronavirus 2 (SARS-CoV-2) has caused the catastrophic COVID-19 pandemic, which has infected > 250 million and caused > 5 million deaths worldwide to date (Johns Hopkins Coronavirus Resource Centre, 10th Nov 2021). Reverse transcription quantitative PCR (RT-qPCR) remained the gold standard diagnostic test for SARS-CoV-2 in many countries, with standard diagnostic protocol from the World Health Organisation. However, this procedure requires processing at a clinical diagnostic laboratory which would take hours or days, depending on the workload at the test site. Given the high infectious rate of SARS-CoV-2 with > 500,000 daily new cases globally, there is an urgent need to develop scalable, low-cost detection methods for SARS-CoV-2 to increase our capacity to perform daily testing.

Loop-mediated isothermal amplification (LAMP) is a one-step nucleic acid amplification method that has been applied for diagnostic of infectious diseases^1^. The LAMP reaction involves auto-cycling strand displacement DNA synthesis, using hairpin-forming LAMP primers which anneal to the target DNA template and a DNA polymerase with strand displacement activity. These annealed LAMP primers are in turn displaced by displacement primers for subsequent amplification and elongation^2^. The LAMP reaction is highly sensitive, specific and only requires one set temperature for nucleic acid amplification, providing an attractive technology for the development of a low cost, point-of-care diagnostics for SARS-CoV-2. Recently, there has been significant development on the use of LAMP for the detection of SARS-CoV-2. Several LAMP assays have been developed that target different regions of SARS-CoV-2, including RNA-dependent RNA polymerase (RdRP)^3^, non-structural protein 3 (nsp3)^4^ and the nucleocapsid (N) gene^5^. However, how these LAMP assays perform relative to each other remain unclear and understanding this would facilitate development of a robust LAMP assay for SARS-CoV-2 detection.

In this study, we perform a comparative analysis of three of the earliest LAMP assays developed for SARS-CoV-2 detection^3–5^. We perform a head-to-head comparison of the sensitivity and kinetics to detect the RNA or cDNA of SARS-CoV-2 genes, as well as their capability to be used as colorimetric or fluorescent detection.

## Materials and methods

### Saliva sample collection

Saliva sample collection was approved by the Human Research Ethics Committee of the Alfred Hospital (740/20) and carried out in accordance with the approved guidelines. Informed consent was obtained from all patients, and the experiments conformed to the principles set out in the WMA Declarations of Helsinki. Saliva samples are collected from 3 healthy patients (Patient 1: 25 years old female, Patient 2: 29 years old male, Patient 3: 25 years old female), using the ‘enhanced saliva’ collection method as previously described for SARS-CoV-2 detection^6^. Briefly, the patients were instructed to sniff strongly to gather any nasal secretion into the oropharynx, cough to produce any phlegm, and submit these secretions and additional saliva into a 50 ml Falcon tube. The saliva samples are processed for RT-LAMP assay right away and the remaining samples are stored at -80C for long term storage.

### Molecular reagents

For positive DNA control, two regions in SARS-CoV-2 ORF1, nt.2945–3370 and nt.14971–15970, were synthesized as gBLOCK double stranded DNA fragments (Integrated DNA Technologies), as well as the 2019-nCoV_N positive control plasmid carrying the SARS-CoV-2 Gene N (GenBank: NC_045512.2, Integrated DNA Technologies).

For positive RNA control, we used the Twist Synthetic SARS-CoV-2 RNA control which provides coverage of > 99.9% of the viral genome in six non-overlapping 5 kb single stranded RNA fragments (GeneBank: MT007544.1, Twist Bioscience).

### RT-LAMP/LAMP colorimetric assay

Positive DNA or RNA controls were diluted to various concentrations and used in LAMP and RT-LAMP reactions respectively. The sequences of primers and controls used are listed in Supplementary table 1. The six primers (F3, B3, FIP, BIP, Loop F and Loop B) were premixed as a 10X working stock containing 2 µM of each outer primer (F3 & B3), 16 µM of each inner primer (FIP & BIP), and 4 µM of each loop primer (Loop F & Loop B).

For colorimetric detection, a 20 μl reaction was set up containing 10 μl of WarmStart Colorimetric LAMP 2X Master Mix (New England Biolabs), 2 μl primer mix, 1 μl RNA/DNA control and 7 μl nuclease-free water. The mixed reactions were incubated at 65 °C using a heat block and pictures for colorimetric changes were taken from 10–45 min. Subsequently, the amplified samples were run on an 1% agarose gel to determine amplicon specificity.

### RT-LAMP fluorescent assay

Same primer sets and positive RNA controls were used for the RT-LAMP colorimetric assay. For fluorescent detection of RT-LAMP, a 20 μl reaction was set up containing 10 μl of WarmStart Colorimetric LAMP 2X Master Mix (New England Biolabs), 2 μl primer mix, 1 μl RNA control, 1 μl of 10X SYBR green (Thermo Fisher) and 6 μl nuclease-free water. The mixed reactions were incubated at 65 °C using a StepOne Plus Real Time PCR machine (Thermo Fisher) for 60 min and fluorescence measurements were taken every 2.5 min.

To quantify the kinetics of the RT-LAMP assay, the ΔRn values are extracted and plotted against the timepoints. A threshold corresponding to half the maximum ΔRn value is set to determine the time required to reach half the maximum fluorescence intensity, termed ‘½ ΔRn_max_’. If no signal was detected after 60 min reaction time, the ½ ΔRn_max_ was listed as 60 min.

### RT-qPCR assay for SARS-CoV-2 detection

RT-qPCR diagnostic assay for SARS-CoV-2 was performed using the CDC 2019-Novel Coronavirus Real-Time RT-PCR Diagnostic Panel for N1 and N2 assays (Research Use only kits, IDT technologies), following the in-vitro diagnostic procedures specified by the US Centers for Disease Control and Prevention (CDC-006-00,019, Revision 6). The GoTaq Probe 1-step RT-qPCR kit (Promega) was used, with the Twist Synthetic SARS-CoV-2 RNA control (Twist Bioscience) as template. Briefly, a 20 μl reaction was set up consisting of 5ul RNA template, 3.1 μl nuclease-free water, 1.5 μl primer mix, 10 μl qPCR master mix, 0.4 μl Go Script RT Mix. RT-qPCR was performed using the StepOne Plus Real Time PCR machine (Thermo Fisher), using the thermal profile: 45 °C for 15 min; 95 °C for 2 min; 45 cycles of 95 °C for 3 s, 55 °C for 30 s. The ΔRn values and cycle numbers were extracted for analysis using the Step One software (Thermo Fisher).

## Results

### Comparative study of LAMP reactions using colorimetric detection

For this study, we used the WarmStart Colorimetric RT-LAMP 2X Master Mix (New England Biolabs) containing a warm-start reverse transcriptase RTx and an isothermal DNA polymerase Bst 2.0, which was capable of both LAMP and RT-LAMP. The reaction mix also contained a pH indicator, which allowed visualisation of amplification as a result of protons produced by the LAMP reaction and led to a red-to-yellow colour change. We selected primer sets from three previous studies that targeted different regions of the SARS-CoV-2 genome (Supplementary table 1). The ‘ORF1ab RdRP’ set^3^ and the ‘ORF1ab nsp3’ set^4^ targeted RdRP and nsp3 sequences encoded by ORF1ab respectively, while the ‘Gene N’ set targeted the nucleocapsid gene^5^.

**Figure 1:**
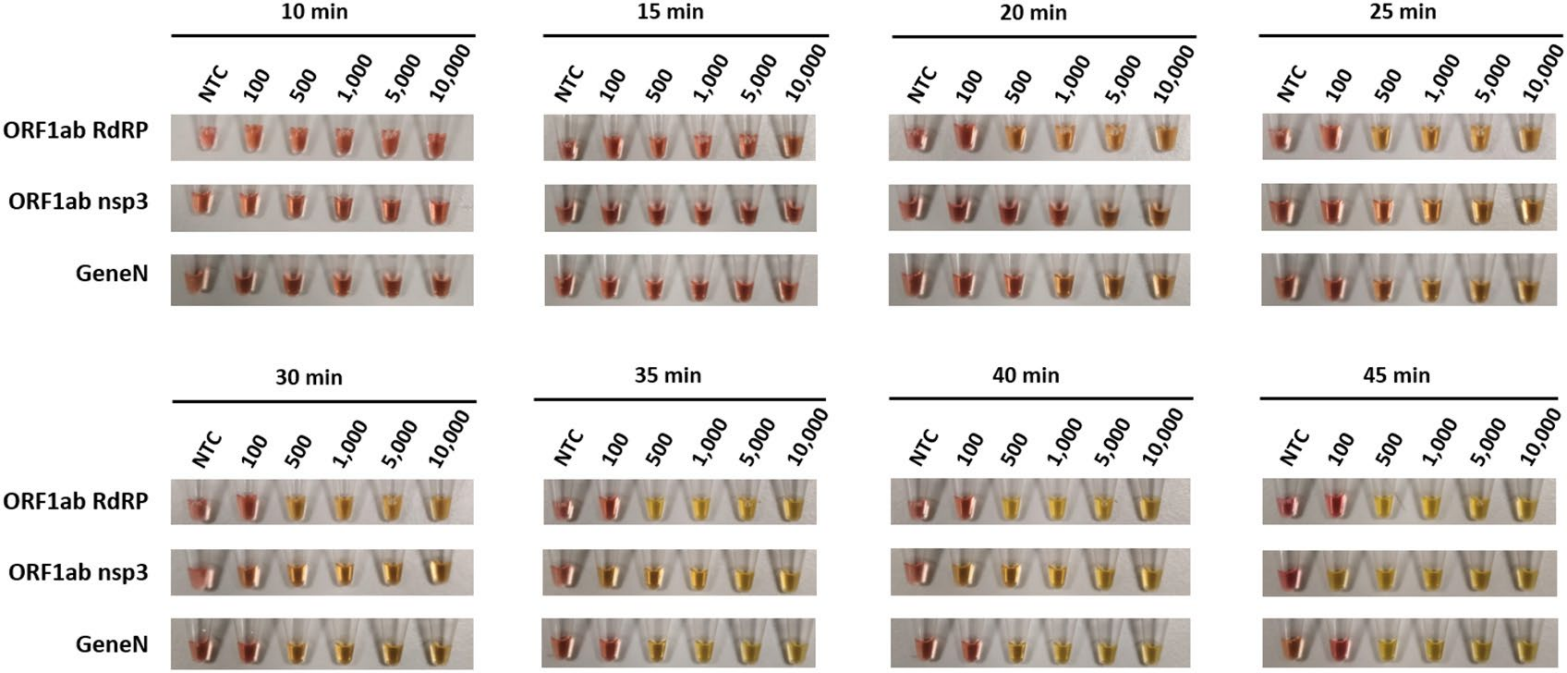
Colorimetric detection of SARS-CoV-2 DNA using LAMP. LAMP reactions are assessed at different time points using a dilution series of positive DNA control ranging from 100 copies to 10,000 copies. NTC (no template control) is used as a negative control. n = 3 biological repeats.

**Figure 2:**
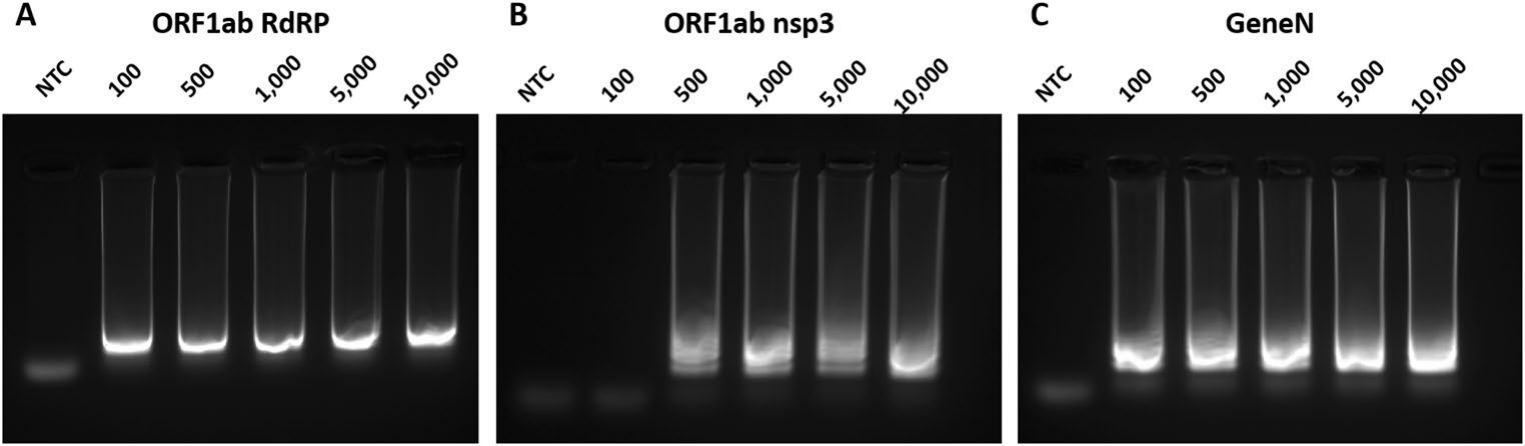
Gel electrophoresis analysis of LAMP assays. Representative gel picture is shown for (**A**) ORF1ab RdRP, (**B**) ORF1ab nsp3 and (**C**) Gene N in LAMP assays, using a dilution series of positive DNA control ranging from 100 copies to 10,000 copies. NTC (no template control) is used as a negative control. N = 3 biological repeats.

We first assessed the capability of these three primer sets in colorimetric LAMP reactions, using synthesized DNA of SARS-CoV-2 genes as control. Our results showed that the three primer sets are all capable of LAMP detection of the SARS-CoV-2 genes, albeit with some differences in performances (Fig. 1). In terms of kinetics for the colorimetric LAMP reaction, visible colour changes could be observed as early as 20 min for ORF1ab RdRP and Gene N, compared to 25 min for ORF1ab nsp3. In order to determine the sensitivity of the LAMP assays, we performed LAMP reactions with a dilution series ranging from 100 to 10,000 copies of DNA molecules. Overall the lowest detection limit was 100 copies of DNA molecules for both ORF1ab nsp3 and Gene N, compared to 500 copies of DNA molecules for ORF1ab RdRP (Figs. 1–2). In addition, gel electrophoresis showed a discrete band representing the LAMP amplicons (Fig. 2A–C), supporting the specificity of the LAMP amplification for all three primer sets. Collectively, all three primer sets are capable of colorimetric LAMP detection of SARS-CoV-2 within 30 min, with slightly faster kinetics for the ORF1ab RdRP/Gene N and slightly higher sensitivity for ORF1ab nsp3.

### Comparative study of RT-LAMP reactions using colorimetric detection

**Figure 3:**
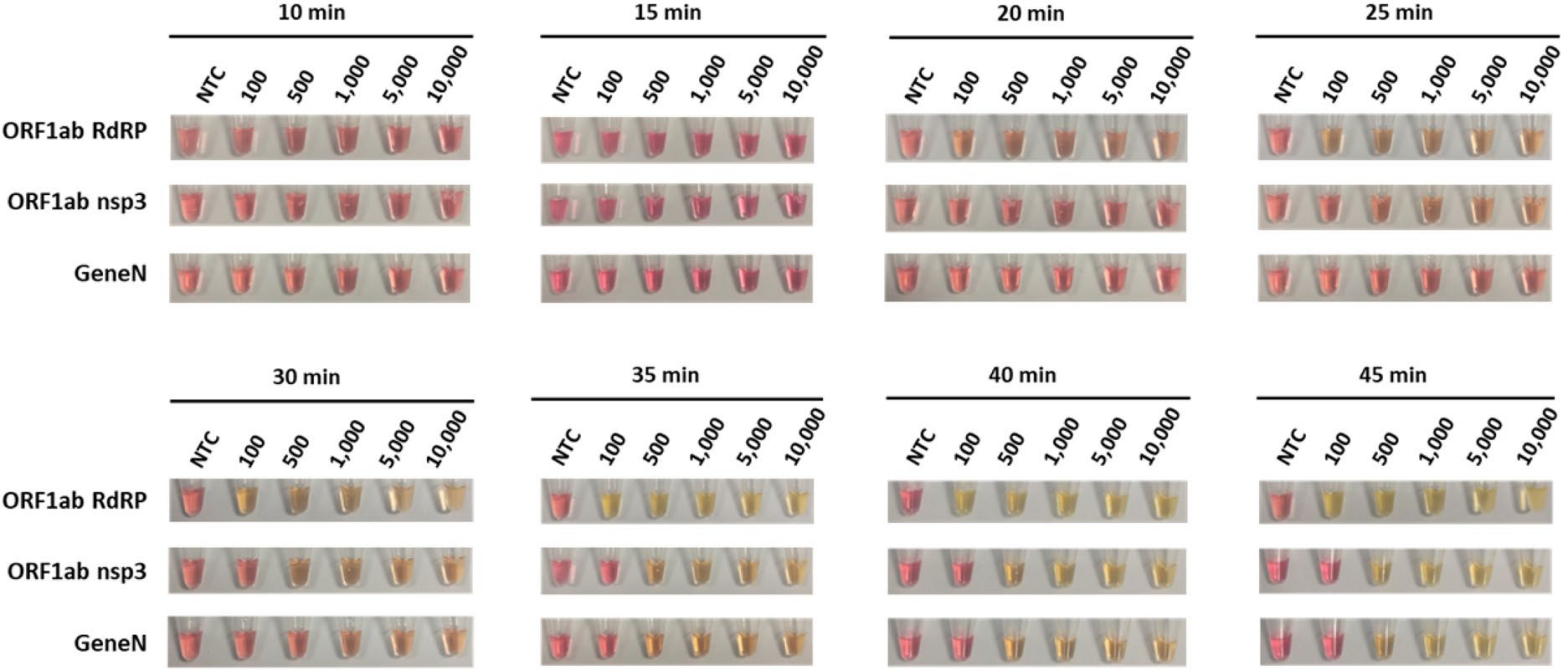
Colorimetric detection of SARS-CoV-2 RNA using RT-LAMP. RT-LAMP reactions are assessed at different time points using a dilution series of positive RNA control ranging from 100 copies to 10,000 copies. NTC (no template control) is used as a negative control. n = 3 biological repeats.

**Figure 4:**
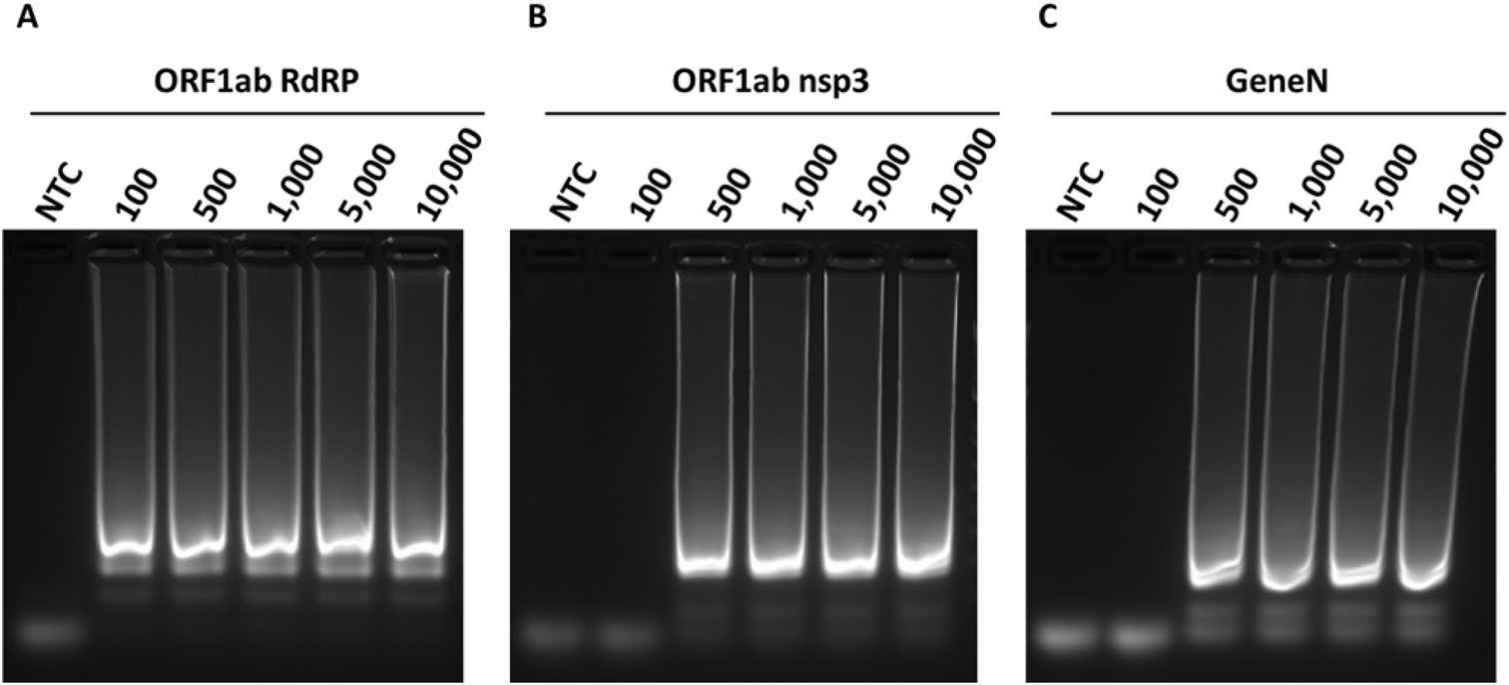
Gel electrophoresis analysis of RT-LAMP assays. Representative gel picture is shown for (**A**) ORF1ab RdRP, (**B**) ORF1ab nsp3 and (**C**) Gene N in RT-LAMP assays, using a dilution series of positive RNA control ranging from 100 copies to 10,000 copies. NTC (no template control) is used as a negative control. n = 3 biological repeats.

Next, we compared the three primer sets in colorimetric RT-LAMP reactions, using in vitro transcribed RNA of SARS-CoV-2 genes as control. Similar to our LAMP results, the ORF1ab RdRP was the fastest out of the three with visible colour change after 20 min of reaction, whereas ORF1ab nsp3 and Gene N took 25 min and 30 min respectively (Fig. 3). In terms of sensitivity, the lowest detection limit was observed in ORF1ab RdRP (100 copies of RNA molecules), followed by ORF1ab nsp3 and Gene N (both 500 copies of RNA molecules, Fig. 3). However for the ORF1ab RdRP and nsp3 primer sets, we noticed that the no template control sometimes exhibited colour change indicative of a false-positive, while this was not observed for the Gene N set (Table 1). Correspondingly, analysis by gel electrophoresis revealed a clear band of the correct RT-LAMP reaction product in lanes with ≥ 100 copies RNA input for ORF1ab RdRP, and ≥ 500 copies RNA input for both ORF1ab nsp3 and Gene N (Fig. 4A–C). These results supported the specificity of the RT-LAMP amplification using the three primer sets. Overall, the three primer sets performed similarly in LAMP or RT-LAMP for colorimetric detection, with only subtle differences in sensitivity and kinetics.

**Table 1:** Summary of false positives detected in no template control in biological repeats (n=5-6).

Primer sets	Assay	False positive after 15 min	False positive after 30 min	False positive after 45 min
ORF1ab RdRP	LAMP	0/6 (0%)	3/6 (50%)	4/6 (66%)
	RT-LAMP	1/5 (20%)	4/5 (80%)	4/5 (80%)
ORF1ab nsp3	LAMP	0/6 (0%)	1/6 (16%)	2/6 (33%)
	RT-LAMP	1/5 (20%)	2/5 (40%)	3/5 (60%)
Gene N	LAMP	0/6 (0%)	0/6 (0%)	3/6 (50%)
	RT-LAMP	0/5 (0%)	0/5 (0%)	0/5 (0%)

### Assessment of the kinetics and sensitivity of RT-LAMP reactions using fluorescent detection

**Figure 5:**
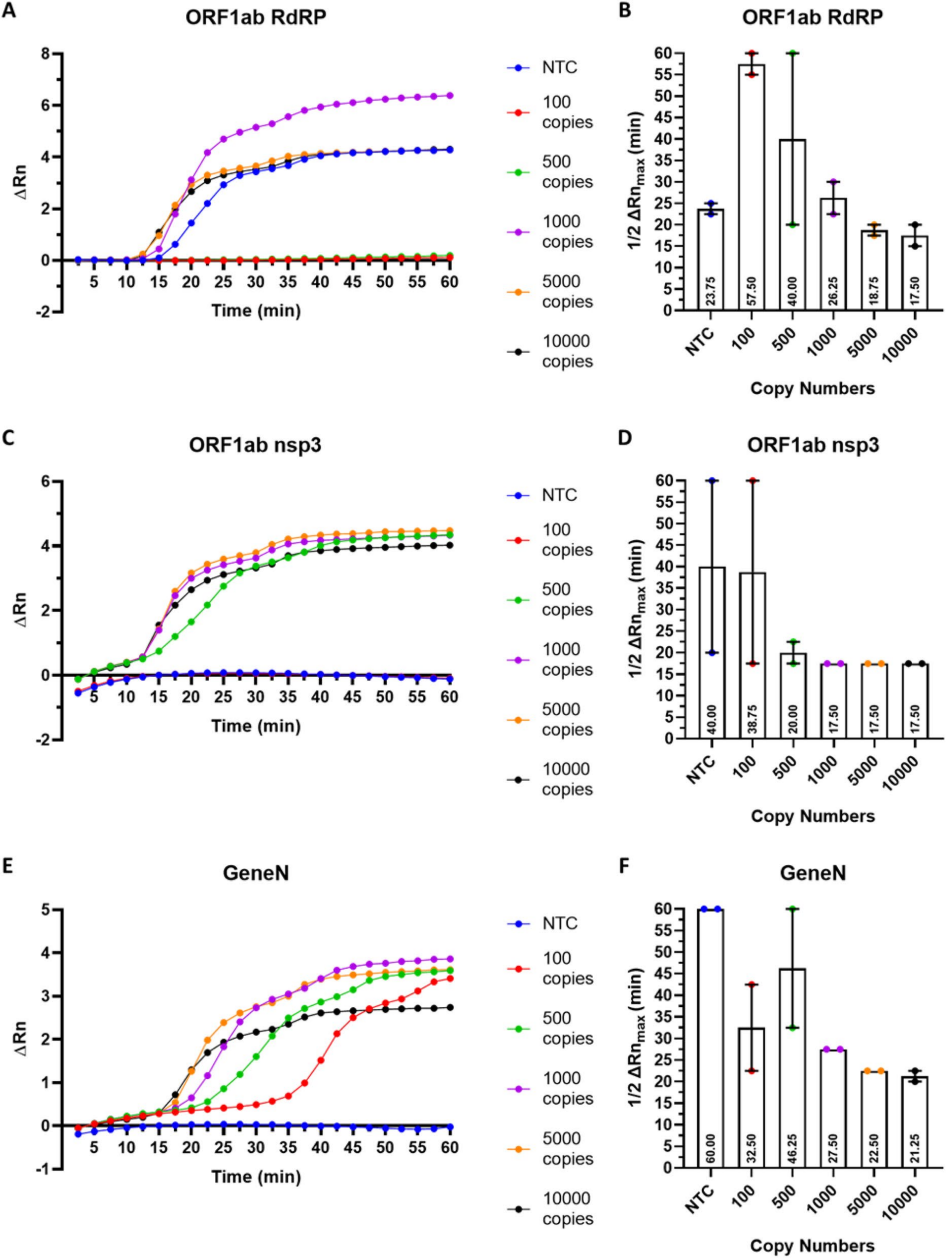
Fluorescent detection of SARS-CoV-2 RNA using RT-LAMP. Real-time measurement of SARS-CoV-2 RNA using RT-LAMP for (**A**) ORF1ab RdRP, (**C**) ORF1ab nsp3 and (**E**) Gene N, showing the normalised fluorescent signal (ΔRn) against the reaction time. Quantification of the time to reach half the maximum fluorescent signal (ΔRn_max_) for (**B**) ORF1ab RdRP, (**D**) ORF1ab nsp3 and (**F**) Gene N. Results are displayed as the mean of 2 biological repeats, error bars represent SEM.

Although colorimetric detection represents an easy detection method for point-of-care diagnostic development, subtle colour changes may not be obvious with naked eye. To more accurately assess the sensitivity and kinetics of the three primer sets, we added SYBR Green I into the RT-LAMP reactions which allows fluorescent quantification using a qPCR machine. For all three primer sets, increase in the fluorescent signal started around 12–15 min. The fluorescent signal reached a plateau around 30–40 min for ORF1ab RdRP and ORF1ab nsp3 (Fig. 5A, C), indicating that 40 min of reaction is sufficient for these two primer sets. However, Gene N exhibited different amplification curves depending on the copy number of the RNA molecules: ~ 30 min should be sufficient for detection of ≥ 500 copies, whereas > 45 min is needed for detection of 100 copies of RNA molecules (Fig. 5E). This supported that the Gene N RT-LAMP has a high sensitivity, capable of detecting as low as 100 copies of SARS-CoV-2 RNA.

**Figure 6:**
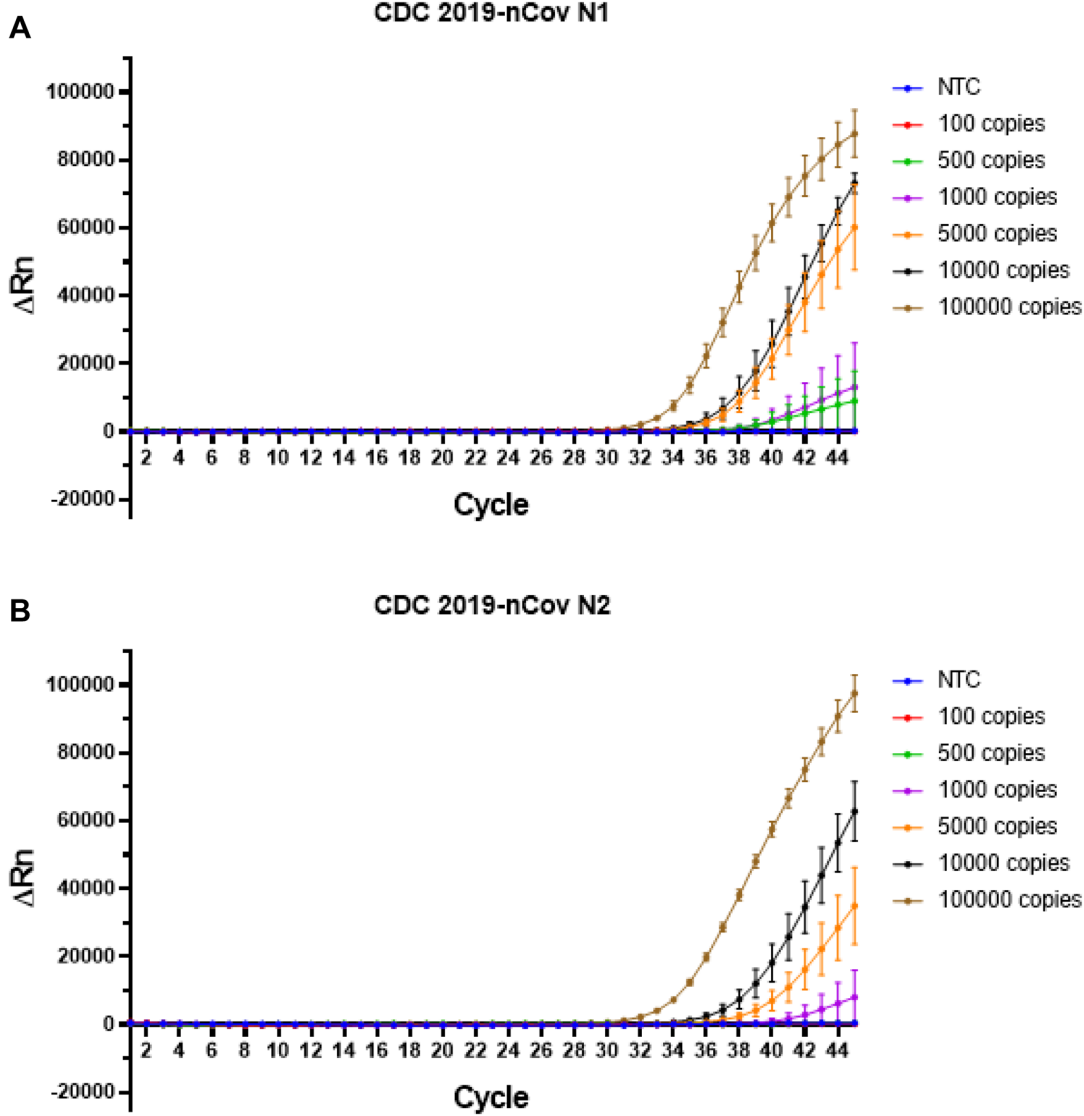
Detection limit of RT-qPCR assay using the CDC real-time RT-PCR diagnostic panel. Real-time measurement of SARS-CoV-2 RNA using the (**A**) 2019-nCov N1 assay and (**B**) 2019-nCov N2 assay was recorded as normalised fluorescent signals (ΔRn) along 45 cycles. Pooled results are displayed as means of six biological and technical repeats, error bars represent SEM.

To better benchmark the performance of RT-LAMP, we compared the sensitivity with the current gold standard RT-qPCR assay for SARS-CoV-2 diagnostics, using the 2019-nCoV real-time RT-PCR diagnostic panel developed by the USA Centre for Disease Control and Prevention (CDC). This diagnostic panel consisted of two primer sets targeting the selected regions of the N gene, termed N1 and N2 assay. Our results showed that both N1 and N2 assays can robustly detect 5000 copies of SARS-CoV-2, with fluorescent signal increase at 35 and 37 cycles respectively (Fig. 6). The lowest detection limit of N1 assay was is 500 copies whereas for the N2 assay is 1000 copies, albeit at high cycle numbers (39 and 41 cycles respectively). In comparison, the Gene N RT-LAMP fluorescent assay can detect as low as 100 copies, suggesting that the RT-LAMP method can outperform RT-qPCR in terms of sensitivity for SARS-CoV-2 RNA detection.

To compare the kinetics of RT-LAMP across the three primer sets, we measured the time required to reach half the maximum fluorescence signal (½ ΔRn_max_). We noted that ½ ΔRn_max_ is generally consistent for detection of ≥ 1000 copies of RNA molecules, whereas detection of 100 or 500 copies of RNA molecules yielded variable ½ ΔRn_max_values in biological repeats. This suggested the three primer sets performed more robustly for detection of higher copies of SARS-CoV-2 RNA. For detection of 1000 copies of RNA molecules, ORF1ab nsp3 exhibited the fastest kinetics (½ ΔRn_max_ = 17.5 min), followed by ORF1ab RdRP (½ ΔRn_max_ = 26.25 min) and Gene N (½ ΔRn_max_ = 27.5 min). However, for detection of 10,000 copies of RNA molecules, all three primer sets exhibited comparable kinetics (½ ΔRn_max_ = 17.5–21.25 min) (Fig. 5B, D, F). Collectively, our results suggested that the three RT-LAMP assays have comparable kinetics for detection of high copies of SARS-CoV-2, but variable performances for detection of low copies of SARS-CoV-2.

### False positive rates for RT-LAMP/LAMP reactions

In our testing, occasionally we detected positive signals in the no template controls which were indicative of false positives. Table 1 listed the false positive rate for the three primer sets in RT-LAMP and LAMP reactions. In general, we observed lower false positive rates in shorter reaction time (15 min) and higher false positive rates in longer reaction time (45 min), thus we recommend using a reaction time of 30 min. Notably in a 30 min RT-LAMP reaction, the lowest false positive rate is observed in Gene N (0/5, 0%), followed by ORF1ab nsp3 (2/5, 40%) and ORF1ab RdRP (4/5, 80%). These results suggested that the Gene N primer set has the best accuracy for detection among the three tested assays.

**Figure 7:**
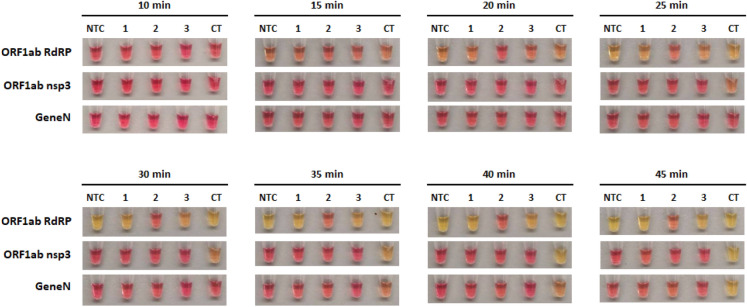
False-positive testing of RT-LAMP in clinical saliva samples. RT-LAMP reactions are assessed at different time points in saliva samples from 3 healthy patients (1–3). NTC indicates no template control as a negative control. CT indicates positive control using Twist Synthetic SARS-CoV-2 RNA (10,000 copies). n = 3 independent experiments.

We have further evaluated the false positive rates for the three RT-LAMP assays in clinical saliva samples (Fig. 7, Table 2), given that saliva samples may contain lots of contaminating RNA derived from cells or bacteria which could lead to false positive detections. Using an ‘enhanced saliva’ collection protocol optimised for SARS-CoV-2 detection 6, we collected saliva samples from 3 healthy patients. Our results showed that in a 45 min RT-LAMP reaction there was no false positive detection for Gene N (0/9, 0%), while ORF1ab nsp3 had 33% false positive rate (3/9) and ORF1ab RdRP had a 78% false positive rate (7/9). Shortening the reaction time to 30 min helped lower the false positive rate of ORF1ab nsp3 (0/9, 0%), but had no effect on ORF1ab RdRP (7/9, 78%). Collectively, our results for RT-LAMP assay using SARS-CoV-2 RNA and clinical saliva samples consistently showed that the Gene N primer set had the best accuracy with zero false positive detection, followed by ORF1ab nsp3 and ORF1ab RdRP.

**Table 2:** Summary of false positives in 3 independent RT-LAMP experiments using saliva samples from healthy patients.

Primer sets	Total false positive rate	Healthy patient	False positive after 15 min	False positive after 30 min	False positive after 45 min
ORF1ab RdRP	7/9 (78%)	Patient 1	0/3 (0%)	3/3 (100%)	3/3 (100%)
		Patient 2	0/3 (0%))	1/3 (33%)	1/3 (33%)
		Patient 3	0/3 (0%)	3/3 (100%)	3/3 (100%)
ORF1ab nsp3	3/9 (33%)	Patient 1	0/3 (0%)	0/3 (0%)	1/3 (33%)
		Patient 2	0/3 (0%)	0/3 (0%)	1/3 (33%)
		Patient 3	0/3 (0%)	0/3 (0%)	1/3 (33%)
Gene N	0/9 (0%)	Patient 1	0/3 (0%)	0/3 (0%)	0/3 (0%)
		Patient 2	0/3 (0%)	0/3 (0%)	0/3 (0%)
		Patient 3	0/3 (0%)	0/3 (0%)	0/3 (0%)

Total false positive rate is calculated for a 45 min RT-LAMP reaction on 9 biological repeats (3 patient samples repeated in 3 independent experiments).

## Discussion

Active cases of SARS-CoV-2 can be diagnosed by detection of the viral RNA or antigens in patient samples. While antigen-based detection methods are cheap and rapid as a point-of-care diagnostics, they are inherently less sensitive compared to viral RNA detection methods given there is no amplification of protein signals. As such, the sensitivity of many antigen-based detection methods ranges from 50 to 90%^7^**.** In contrast, viral RNA detection methods, such as RT-qPCR remain the gold standard for SARS-CoV-2 diagnostics with ~ 98% sensitivity. However, the requirement of sample processing in a clinical diagnostic laboratory results in long turnaround time for diagnostic results and this remains the key bottleneck to upscale our capability to perform daily SARS-CoV-2 testing.

Recent development of RT-LAMP assays could address this key issue, providing a rapid and low-cost detection method for SARS-CoV-2. In this study, we compared and validated three LAMP-based assays previously developed to detect different parts of SARS-CoV-2: ORF1ab RdRP, ORF1ab nsp3 and Gene N. Our results show that the three RT-LAMP assays allow colorimetric detection of SARS-CoV-2 genes in 30 min, with robust accuracy at detecting 1000 RNA copies. Also, the three assays performed similarly for detection of RNA or DNA copies of SARS-CoV-2. We show that the sensitivity of RT-LAMP can be further improved when coupled with fluorescence detection. This allows detection of as little as 100 RNA copies using the Gene N assay, representing a higher sensitivity to the CDC 2019-nCoV RT-qPCR assays. However, we observe some variability in these RT-LAMP assays with shorter reaction time and detection of lower load of SARS-CoV-2 RNA. Notably, in our testing we observe false positive results in some experimental repeats in non-template control and clinical saliva samples from healthy patients. Our RT-LAMP results in non-template controls and clinical saliva samples consistently show that the Gene N has the best accuracy with zero false positive detection, follow by ORF1ab nsp3 (33–60%) and ORF1ab RdRP (78–80%). Our study highlights the importance of optimal primer design to decrease false-positives in RT-LAMP detection of SARS-CoV-2. Overall for RT-LAMP, our comparative analysis show that the ORF1ab RdRP and ORF1ab nsp3 sets have faster kinetics for detection, whereas the Gene N primer set exhibits no false positives in 30 min reaction time. Future optimisation of the primer sets and modification of RT-LAMP reactions would be important to develop an accurate and sensitive detection method for SARS-CoV-2, such as the use of quenched fluorescent primers to reduce the false positive rates of RT-LAMP^8^. There are also ongoing development of new RT-LAMP-based assays to improve performance of SARS-CoV-2 detection, including the use of a two-stage isothermal amplifications (Penn-RAMP)^9^, integration of CRISPR with RT-LAMP^10,11^, as well as barcoded RT-LAMP reactions to allow high-throughput processing using next-generation sequencing (LAMP-seq)^12^. Future validation of these new RT-LAMP-based assays would be important to translate these technologies into clinical testing in the real world.

Compared to RT-qPCR detection of SARS-CoV-2, there are several key advantages of the RT-LAMP detection method. The major advantage is the rapid reaction time of ~ 30 min, which is far superior compared to RT-qPCR with a turn-around time of several hours to days. Secondly, the readout of RT-LAMP is simple and can be interpreted easily by the naked eye, either as a change in colour (this study) or turbidity^13^. Finally, RT-LAMP only requires one isothermal step which makes it simple to develop low-cost, portable devices to process the samples. Recently, several companies have developed portable devices to carry out point-of-care RT-LAMP testing, such as DNAfit LifeSciences (UK) and Lucira Health (USA). Moreover, a recent study also demonstrated that RT-LAMP can be successfully conducted in a kitchen range oven, providing a low cost solution to carry out RT-LAMP testing at home^14^. Further improvement to RT-LAMP would potentially provide an affordable point-of-care detection method for routine SARS-CoV-2 detection.

## Conclusions

In summary, this study shows a detailed assessment of the performances of LAMP-based assays for detection of SARS-CoV-2, providing important validation data that would facilitate further development of LAMP-based diagnostics for SARS-CoV-2 and translation into real world testing.

## Supplementary Information

Supplementary Information 1.

Supplementary Information 2.
