# Supplementary material for: Comparative analysis of loop-mediated isothermal amplification (LAMP)-based assays for rapid detection of SARS-CoV-2 genes
Source: Sci Rep. 2021 Nov 18;11:22493. doi: 10.1038/s41598-021-01472-3 (PMC8602269; doi:10.1038/s41598-021-01472-3)
Supplement: Supplementary file 1 — Supplementary Information 1. [file 41598_2021_1472_MOESM1_ESM.pdf]

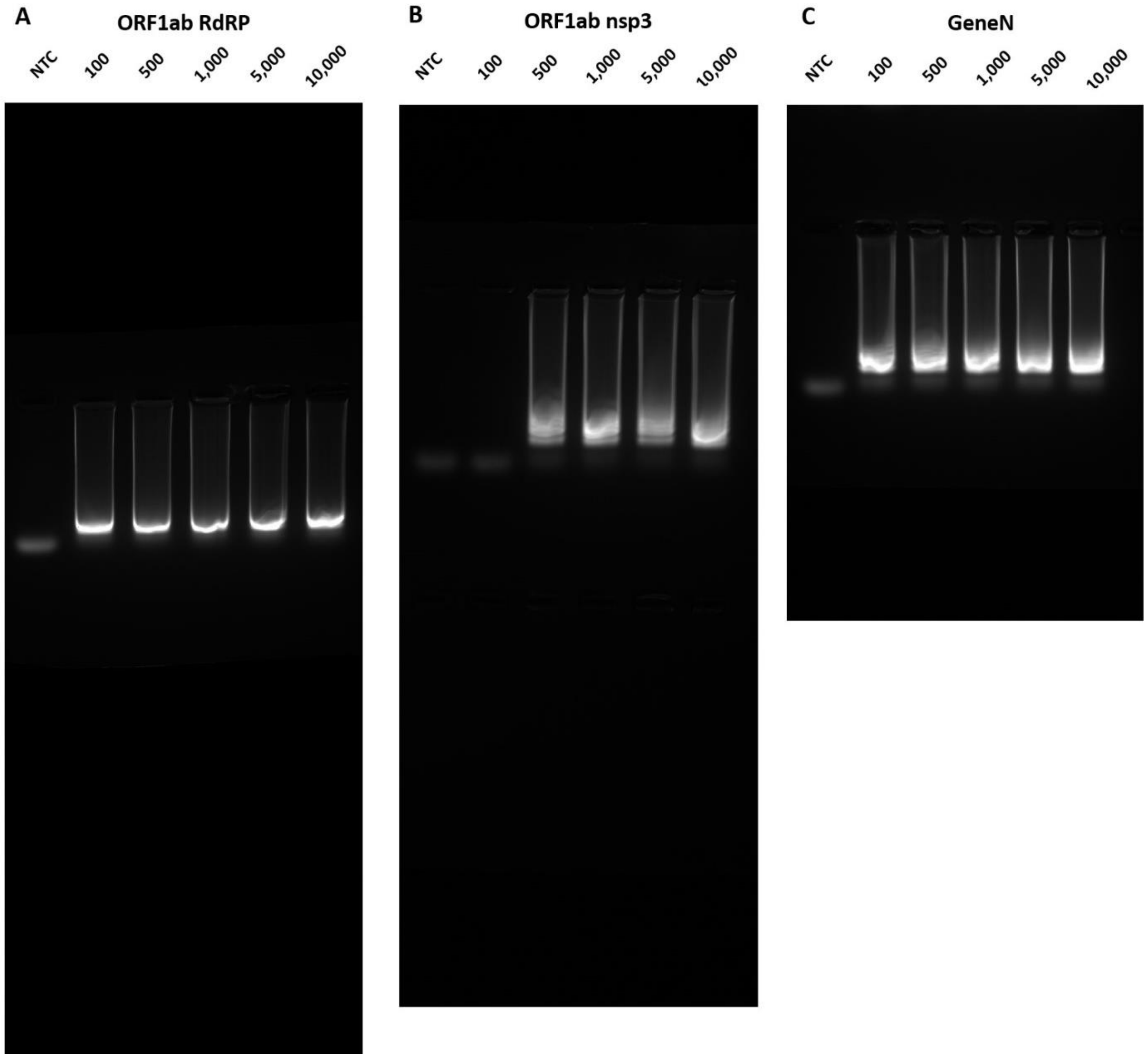


**Full gel picture of Figure 2: Gel electrophoresis analysis of LAMP reaction. LAMP reaction for ORF1ab RdRP, ORF1ab nsp3 and GeneN are run on different gels.**

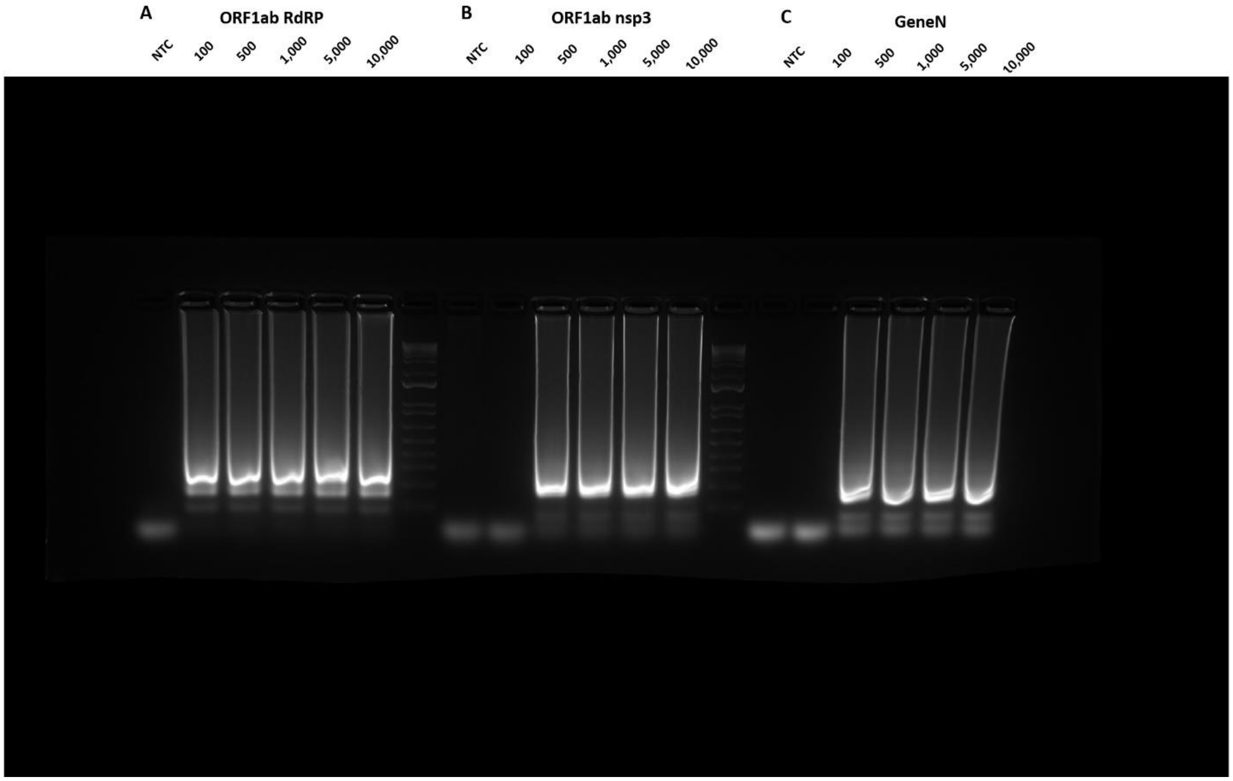


**Full gel picture of Figure 4: Gel electrophoresis analysis of RT-LAMP reaction.**
