# Supplementary material for: Comparative analysis of loop-mediated isothermal amplification (LAMP)-based assays for rapid detection of SARS-CoV-2 genes
Source: Sci Rep. 2021 Nov 18;11:22493. doi: 10.1038/s41598-021-01472-3 (PMC8602269; doi:10.1038/s41598-021-01472-3)
Supplement: Supplementary file 2 — Supplementary Information 2. [file 41598_2021_1472_MOESM2_ESM.pdf]

Supplementary Information

Comparative analysis of loop-mediated isothermal amplification (LAMP)-based assays for rapid detection of SARS-CoV-2 genes

Daniel Urrutia-Cabrera, Roxanne Hsiang-Chi Liou, Jiang-Hui Wang, Jianxiong Chan, Sandy Shen-Chi Hung, Alex W Hewitt, Keith R Martin, Thomas L Edwards, Patrick Kwan, Raymond Ching-Bong Wong

Supplementary table 1: Information of primers and control gBLOCK used in this study.

Primer	Sequence	Reference
RW244 nsp3 F3	TCCAGATGAGGATGAAGAAGA	[4]
RW245 nsp3 B3	AGTCTGAACAACTGGTGTAAG	[4]
RW246 nsp3 FIP(F1c+F2)	AGAGCAGCAGAAGTGGCACAGGTGATTGTGAAGAAGAAGAG	[4]
RW247 nsp3 BIP(B1c+B2)	TCAACCTGAAGAAGAGCAAGAACTGATTGTCCTCACTGCC	[4]
RW248 nsp3 LoopF	CTCATATTGAGTTGATGGCTCA	[4]
RW249 nsp3 LoopB	ACAAACTGTTGGTCAACAAGAC	[4]
RW250 RdRp F3	CCACTAGAGGAGCTACTGTA	[3]
RW251 RdRp B3	TGACAAGCTACAACACGT	[3]
RW252 RdRp FIP	AGGTGAGGGTTTTCTACATCACTATATTGGAACAAGCAAATTCTATGG	[3]
RW253 RdRp BIP	ATGGGTTGGGATTATCCTAAATGTGTGCGAGCAAGAACAAGTG	[3]
RW254 RdRp LF	CAGTTTTTAACATGTTGTGCCAACC	[3]
RW255 RdRp LB-4	TAGAGCCATGCCTAACATGCT	[3]
RW256 Gene N-A-F3	TGGCTACTACCGAAGAGCT	[5]
RW257 Gene N-A-B3	TGCAGCATTGTTAGCAGGAT	[5]
RW258 Gene N-A-FIP	TCTGGCCCAGTTCCTAGGTAGTCCAGACGAATTCGTGGTGG	[5]
RW259 Gene N-A-BIP	AGACGGCATCATATGGGTTGCACGGGTGCCAATGTGATCT	[5]
RW260 Gene N-A-LF	GGACTGAGATCTTTCATTTTACCGT	[5]
RW261 Gene N-A-LB	ACTGAGGGAGCCTTGAATACA	[5]
**Positive controls**	**Sequence**	**References**
RWgb80 ORF1ab RdRp 14971-15970	aaggctagactttattatgattcaatgagttatgaggatcaagatgcacttttcgcatatacaaaacgtaatgtcatccctactataactcaaatgaatcttaagtatgccattagtgcaaagaatagagctcgcaccgtagctggtgtctctatctgtagtactatgaccaatagacagtttcatcaaaaattattgaaatcaatagccgccactagaggagctactgtagtaattggaacaagcaaattctatggtggttggcacaacatgttaaaaactgtttatagtgatgtagaaaaccctcaccttatgggttgggattatcctaaatgtgatagagccatgcctaacatgcttagaattatggcctcacttgttcttgctcgcaaacatacaacgtgttgtagcttgtcacaccgtttctatagattagctaatgagtgtgctcaagtattgagtgaaatggtcatgtgtggcggttcactatatgttaaacc	This study
RWgB81 ORF1ab nsp3 2945-3370	ggcattgatttagatgagtggagtatggctacatactacttatttgatgagtctggtgagtttaaattggcttcacatatgtattgttctttctaccctccagatgaggatgaagaagaaggtgattgtgaagaagaagagtttgagccatcaactcaatatgagtatggtactgaagatgattaccaaggtaaaccttggaatttggtgccacttctgctgctcttcaacctgaagaagagcaagaagaagattggttagatgatgatagtcaacaaactgttggtcaacaagacggcagtgaggacaatcagacaactactattcaaacaattgttgaggttcaacctcaattagagatggaacttacaccagttgttcagactattgaagtgaatagttttagtggttatttaaaacttact	This study
